# Prevalence and time trends of spina bifida in fourteen cities located in the Liaoning province of northeast China, 2006-2015

**DOI:** 10.18632/oncotarget.14848

**Published:** 2017-01-27

**Authors:** Gen Ba, Qi-Jun Wu, Yan-Ling Chen, Yan-Hong Huang, Ting-Ting Gong

**Affiliations:** ^1^ Department of Orthopaedics, Shengjing Hospital of China Medical University, Shenyang, China; ^2^ Department of Clinical Epidemiology, Shengjing Hospital of China Medical University, Shenyang, China; ^3^ Liaoning Women and Children's Health Hospital, Shenyang, China; ^4^ Department of Science and Education, Shenyang Women and Children Health Care Centre, Shenyang, China; ^5^ Department of Obstetrics and Gynecology, Shengjing Hospital of China Medical University, Shenyang, China

**Keywords:** Liaoning Province, prevalence, spina bifida, time trend

## Abstract

The present study sought to assess the time trends of spina bifida on the basis of cases identified by the Liaoning Birth Defects Registry in 14 cities from 2006 to 2015. We calculated the prevalence of spina bifida, percent and average change of time trends, and contribution rates of each city. Poisson regression model was used to find the line of best fit for spina bifida prevalence by year, with year as a continuous independent variable. From 2006 to 2015, a total of 2,029 spina bifida cases were identified from 3,248,954 live births (6.25/10,000 live births). We observed statistically significant decreasing trend of overall time trend (11.57% each year). Chaoyang, Fuxin, and Huludao were the top three leading cities, with 14.30/10,000 live births, 9.70/10,000 live births, and 9.20/10,000 live births, respectively. Inversely, the bottom three cities with lowest prevalence were Anshan (2.64/10,000 live births), Dandong (3.43/10,000 live births), and Dalian (3.45/10,000 live births). Of note, we observed significant decreasing trends in over half of these cities (*n* = 8). In addition, the decreasing trend of overall time trend could be mainly attributed to cities of Shenyang, Fushun, and Jinzhou which accounted for nearly one third. In summary, our study suggested a decreasing time trend of spina bifida during the past decade in the Liaoning province. The findings of this study provide evidence that the nationwide folic acid supplement program has been an effective strategy to prevent spina bifida.

## INTRODUCTION

Spina bifida is a type of neural tube defect resulting from an incomplete closure of the spinal column leading to a herniation or exposure of the spinal cord or meninges. Notably, spina bifida is the most common birth defect that can cause disablement for a lifetime [1, 2]. Although a lower fatality rate, approximately 7%, for spina bifida has been observed when compared with the high fatality rate of encephalocele (46%) and anencephaly (100%) [3], spina bifida is associated with long-term physical and cognitive disabilities, which has been a worldwide public health burden [4, 5]. Numerous studies have extensively investigated the epidemiological characteristics of this disease. More specifically, a combination of genetic and environmental factors including family history [6, 7], pre-gestational diabetes [6, 8], maternal obesity [6, 9, 10], insufficient intake of folic acid [6, 11–13], and use of anticonvulsant medications [14, 15] have been established as characteristics of spina bifida. For example, mandatory folic acid fortification of staple cereal grains has been shown to dramatically reduce the risk of pregnancies complicated with spina bifida in many countries [11].

Recently, the prevalence of spina bifida varies by time and region [16]. For instance, on the basis of the National Birth Defects Prevention Network, Parker et al. [17] reported that the prevalence of spina bifida in the United States from 2004 to 2006 was 3.5 per 10,000 live births. In contrast, the prevalence of spina bifida was 42.8 per 10,000 live births in Algeria during the same observational period [16, 18]. Additionally, Onrat et al. [19] suggested that the incidence rate of spina bifida in Turkey from 2003 to 2004 was 19.7 per 10,000 live births. However, these studies based on the data a decade ago which demonstrated great variability in the reported prevalence. In 2002 to 2004, the prevalence of spina bifida was 38.9 per 10,000 live births in Luliang Prefecture, China [5]. However, Li et al. [20] reported that the prevalence was 0.3 per 10,000 live births in Beijing during 2003 to 2009. Although Liu et al. [5] presented a continuous decreasing trend from a peak of 120.0 per 10,000 live births in 2004 to a low of 31.5 per 10,000 live births in 2014, a similar report demonstrated that the time trends and prevalence of spina bifida in China on the basis of the data from the recent decade has been extremely limited. Unknowns have still remained in the prevalence of this malformation during the recent decade. Additionally, we have no idea whether similar decreasing trend could be observed in the other cities. However, recently, there has no formal assessment of the population of Liaoning province which covers an area of 145,900 square kilometers and has a population of almost 42 million. Therefore, to address these aforementioned research questions, we carried out this cross-sectional study by evaluating spina bifida prevalence among infants in Liaoning province from 2006 to 2015.

## RESULTS

The number of live births in 14 cities located in Liaoning province from 2006 to 2015 was demonstrated in Supplementary Table 1. We observed the highest number of live births in 2014 (364,400) but the lowest in 2015 (298,437). Furthermore, Shenyang had the largest number of live births in each year but Benxi had the smallest.

**Figure 1:**
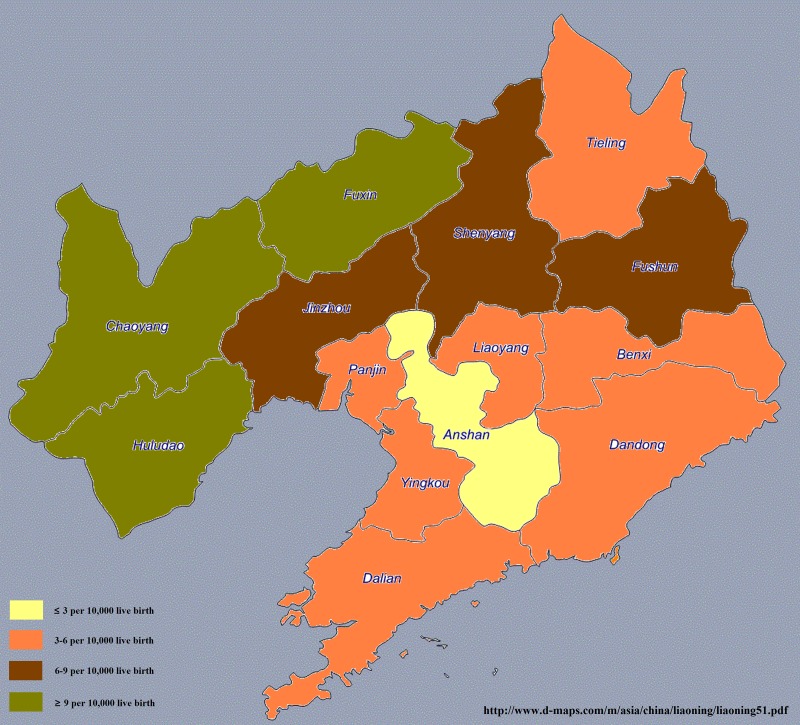
Prevalence of spina bifida in each city of the Liaoning province from 2006 to 2015

The prevalence of spina bifida in each city in the Liaoning province is demonstrated in Figure 1 and Supplementary Table 2. From 2006 to 2015, a total of 2029 spina bifida cases were found (prevalence = 6.25 per 10,000 live births). Notably, the top three leading cities in the Liaoning province were Chaoyang (14.30 per 10,000 live births), Fuxin (9.70 per 10,000 live births), and Huludao (9.20 per 10,000 live births) but the bottom three cities were Anshan (2.64 per 10,000 live births), Dandong (3.43 per 10,000 live births), and Dalian (3.45 per 10,000 live births).

**Figure 2:**
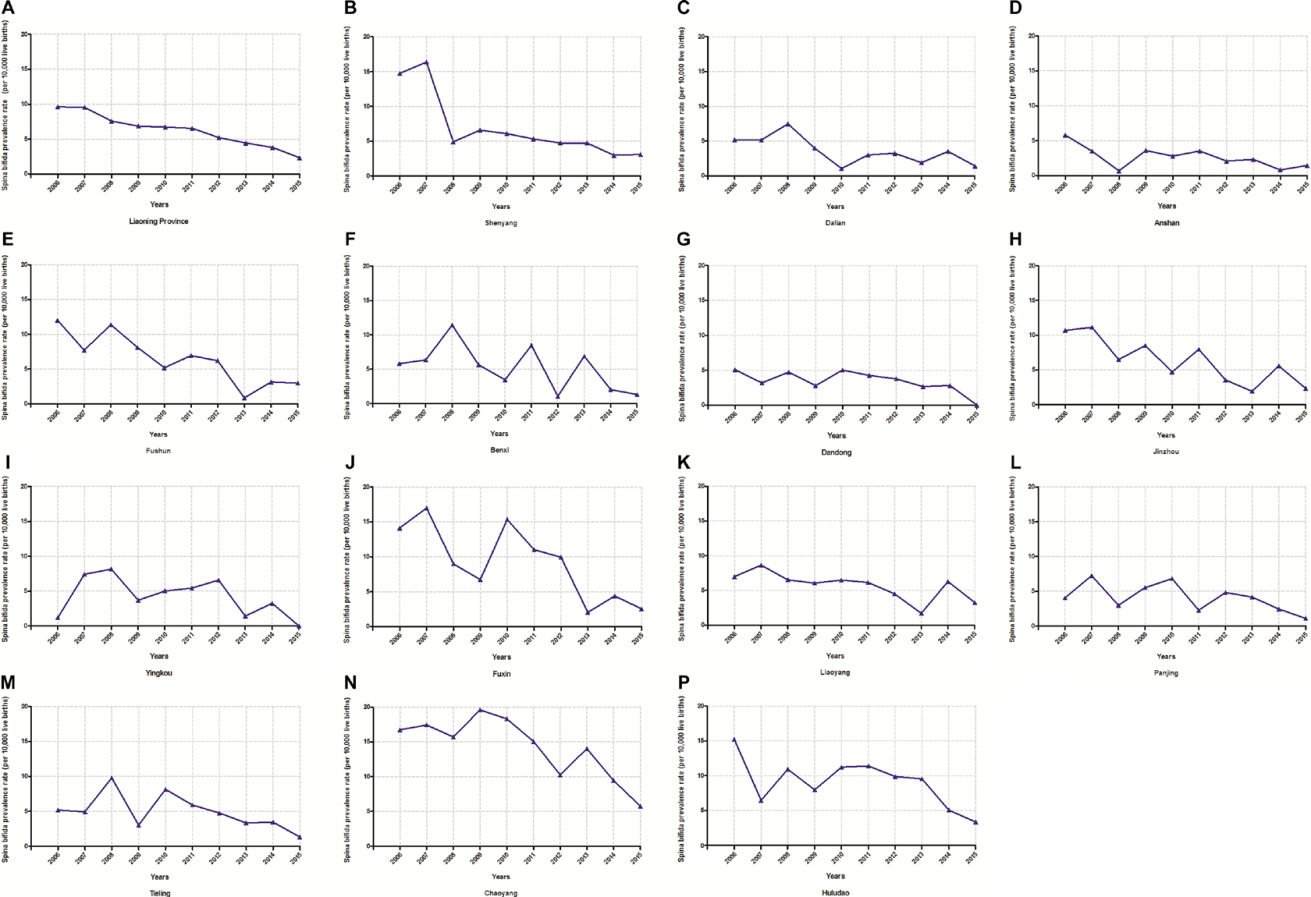
Trends in spina bifida prevalence (per 10,000 live births) of each city in the Liaoning province from 2006 to 2015 (**A**) Liaoning province; (**B**) Shenyang; (**C**) Dalian; (**D**) Anshan; (**E**) Fushun; (**F**) Benxi; (**G**) Dandong; (**H**) Jinzhou; (**I**) Yingkou; (**J**) Fuxin; (**K**) Liaoyang; (**L**) Panjing; (**M**) Tieling; (**N**) Chaoyang; (**P**) Huludao.

The rate and time trend of spina bifida prevalence in each city of Liaoning province from 2006 to 2015 was presented in Figure 2. The overall prevalence decreased from 9.62 to 2.31 per 10,000 live births, with 11.57% per year (*P* < 0.01) (Table 1). When stratifying by cities, we observed decreasing trends in all cities. With the exception of Benxi, Dandong, Yingkou, Panjing, Tieling, and Huludao, the results of the other cites showed statistical significance for the decreasing trend (Table 1). Of note, the decreasing trend of spina bifida prevalence in the Liaoning province could be mainly attributed to cities of Shenyang, Fushun, and Jinzhou which accounted for almost one third (Table 1).

**Table 1:** Trends in spina bifida prevalence in each city of Liaoning during 2006–2015

Spina bifida	2006		2015		PC^†^ (%)	AC^†^ (%)	*P* value	95% CI	Contribution rate (%)
	Case	Rate*	Case	Rate*					
Overall	295	9.62	69	2.31	−75.96	−11.57	< 0.01	−14.19, −8.88	N/A
Shenyang	77	14.74	20	3.07	−79.16	−17.63	< 0.01	−23.49, −11.33	13.37
Dalian	20	5.16	8	1.39	−73.11	−11.13	0.03	−19.71, −1.64	8.13
Anshan	17	5.81	3	1.44	−75.16	−12.89	0.02	−21.66, −3.14	9.51
Fushun	14	12.01	3	2.96	−75.35	−14.10	< 0.01	−21.13, −6.45	10.48
Benxi	5	5.80	1	1.31	−77.40	−8.70	0.22	−22.13, 7.05	6.27
Dandong	8	5.09	0	0.00	−100.00	−4.97	0.13	−11.32, 1.83	3.51
Jinzhou	26	10.70	4	2.36	−78.00	−12.98	0.01	−20.09, −5.23	9.58
Yingkou	2	1.18	0	0.00	−100.00	−6.48	0.36	−20.24, 9.65	4.62
Fuxin	20	14.13	3	2.55	−81.93	−11.75	0.04	−21.54, −0.74	8.61
Liaoyang	9	6.98	3	3.24	−53.56	−6.85	0.05	−13.08, −0.18	4.89
Panjing	4	4.05	1	1.09	−73.12	−7.69	0.17	−18.12, 4.07	5.51
Tieling	11	5.17	2	1.31	−74.68	−7.96	0.18	−19.11, 4.72	5.72
Chaoyang	48	16.74	15	5.75	−65.65	−7.50	0.02	−12.89, −1.79	5.38
Huludao	34	15.23	6	3.37	−77.85	−6.20	0.14	−14.27, 2.63	4.42

Abbreviations: AC, average change; CI, confidence interval; N/A, not available; PC, percent change.

*Spina bifida prevalence was expressed as per 10,000 live births.

^†^Percent change and average change between 2006 and 2015 was calculated by the spina bifida prevalence.

## DISCUSSION

To our knowledge, the present study is one of the limited forms of documentation from China evaluating the time trend of spina bifida prevalence using the data from the recent decade. During the observational period, we observed significant decreasing trend of spina bifida in the Liaoning province from 9.62 per 10,000 live births to 2.31 per 10,000 live births. In addition, decreasing trends were observed in all 14 cities; nevertheless, not all of these cities exhibited statistical significance.

The present study demonstrated the overall prevalence of spina bifida during the 10 years period was 6.25 per 10,000 live births. Zaganjor et al. [16] carried out a systematic review describing the prevalence of spina bifida worldwide which suggested that different areas of different countries had great variation in the prevalence of this defect. The prevalence of spina bifida in the present study was intermediate between the higher prevalence reported in Jordan (59.0/10,000) by Aqrabawi et al. [21] in 2010 and the lower prevalence reported in Spain (0.9/10,000) by European Surveillance of Congenital Anomalies (EUROCAT) [22] in 2012. Moreover, when stratified by income level of country, Zaganjor et al. [16] found a general decrease in the median prevalence for spina bifida from the lower-middle to high income countries. Additionally, the prevalence was intermediate between the higher prevalence reported in Shanxi province (38.9/10,000) by Chen et al. [21] in 2009 and the lower prevalence reported in Beijing (0.3/10,000) by Li et al. [22] in the same year.

Since folic acid supplementation or food fortification is one of the best public strategies to prevent a spina bifida, termination of pregnancy for fetal anomaly is certainly not an optimal solution for this malformation [23]. As the first two countries require mandatory fortification of enriched cereal grain products with 140 μg of folic acid per 100 g, many studies from Canada and the United States have observed a reduction in these malformations after this policy [11]. As of mid-2012, approximately 2.2 billion people were affected after mandatory or voluntary programs were carried out in sixty-seven countries [11, 24]. Nevertheless, the regional differences of prevalence might be attributed to uncommon fortification in Asia and Europe [16]. In 2009, China initiated a nationwide folic acid supplementation program which provided folic acid supplements, free of charge, to all women who have a rural registration as well as who plan to become pregnant [25]. Interestingly, the prevalence of spina bifida in 2012 to 2015 decreased dramatically from that of 2009 in Liaoning province which may be partly due to the effects of this national policy. However, since the varied development of cities and the notion that less women began to take folic acid supplements before pregnancy in the rural area in northern China, not all of the 14 cities showed a statistically significant decreasing trend [26]. Hence, it is imperative that policymakers should focus on this issue in the future.

Several strengthens should be mentioned. The data of this population-based birth defects registry have been in good quality control [27]. Of note, the present study not only described the time trend and prevalence of spina bifida in all 14 cities of Liaoning province but also had a relatively longer observational period. However, limitations of our study include the following. First, we were unable to access the detail information (e.g., demographic or clinical factors) of all live births including cases of spina bifida, which has hindered our ability to focus on the potential causes for the trends. Secondly, because of the access permission, we failed to obtain the prevalence data before 2006 which limited us to investigate whether mandatory premarital physical check-ups, which became voluntary in China since October 1, 2003, play a role in the time trend of spina bifida [5]. Thirdly, the prevalence of spina bifida might be slightly overestimated since we set the denominator of the prevalence of spina bifida as the total number of births which included live births as well as still birth ≥ 28 weeks without induced and spontaneous fetal deaths ≤ 28 weeks [28]. Fourthly, under-reporting may occur in the Shenyang Women and Children Health Care Center compared to these systems in many developed countries since our center began collecting information in 1992 and were vulnerable to several common technical problems that could jeopardize the quality of the collected data [29]. Lastly, since the seventh day after birth was set as the maximal diagnosis time for spina bifida cases [27]. We failed to include sixty-six cases of spina bifida in this study which were confirmed after the seventh day. Herein, the calculated prevalence of spina bifida may be slightly lower than that which includes longer periods.

In conclusion, the present study with population-based designed evaluates the time trend and prevalence of spina bifida in Liaoning province from 2006 to 2015. The findings of this study provide evidence that the nationwide folic acid supplement program has been an effective strategy to prevent spina bifida. However, the folic acid supplement program has limited effectiveness when compared to the mandatory folic acid fortification. Notably, we still observe higher prevalence of spina bifida in some cities of Liaoning province which suggests that the efficiency of the periconceptional folic acid supplementation in pregnancy should be improved.

## MATERIALS AND METHODS

### Study population and data source

The Liaoning Women and Children's Health Hospital is one of the primary obstetrical and gynecological hospitals for the Liaoning province as well as a comprehensive care institution and has been mainly responsible for the healthcare and guidance for women and children of Liaoning. The maternal and child health certificate registry of Liaoning province which provided data from 2006 to 2015 was maintained by this hospital. This registry not only covers all 14 cites of the Liaoning province (Shenyang, Dalian, Anshan, Fushun, Benxi, Dandong, Jinzhou, Yingkou, Fuxin, Liaoyang, Panjing, Tieling, Chaoyang, and Huludao) but also set hospital-delivered live birth and stillbirth infants as monitored subjects. The seventh day after birth was set as the maximal diagnosis time for a congenital malformation case [27].

The procedural details of data collection were previously described [27]. Briefly, we used a ‘Birth Defects Registration Form’ to collect the related information on the infants with spina bifida. To complete the aforementioned form, a trained obstetric or pediatric specialist interviewed the mother of the infant, once the monitor hospital identified and confirmed a spina bifida. Subsequently, the ‘Birth Defects Registration Form’ was first submitted to the local maternal and child health facility and then to Liaoning Women and Children's Health Hospital. A group of state-level experts in medical genetics and pediatrics reviewed and confirmed the data of these cases [27].

Spina bifida is defined as a bony defect of the spine with exposure of meninges and/or neural tissue [23], according to the World Health Organization's International Classification of Diseases, 10th Revision. As such, we included all isolated, multiple cases of spina bifida in the analyses. We requested case ascertainment after termination or examination for these suspected spina bifida cases. Hence, we identified t3,248,954 live births and wo thousand and forty-nine cases of spina bifida during the observational period.

The data quality control was previously described in detail [27]. In brief, according to the program manual to ensure high quality data, the disease diagnosis, data collection, data checking, and medical records were verified by the expert group at each level. In addition, an independent retrospective survey was organized by the experts to find deficiencies and inaccuracies in the data [27].

### Statistical analysis

The birth prevalence of spina bifida was expressed per 10,000 live births. The denominator was based exclusively on the total number of live births. Spina bifida prevalence was calculated for nine 1-year time intervals from 2006 to 2015. To look specifically at time trends, the Poisson regression model was used to find the line of best fit for spina bifida prevalence by year, with year entered into the model as a continuous independent variable [29–32]. The 95% confidence interval (CI) of the average change was calculated by the methods for population-based cancer statistics recommended by the National Cancer Institute [33]. We also calculated the relative contributions for rate changes, which are used for determining the contributions individual cities made to the overall trend [29–34]. All analyses were conducted using SPSS for Windows (version 22, SPSS Inc, Chicago, IL, USA). Two-sided *P* values < 0.05 were considered statistically significant unless otherwise specified.

## SUPPLEMENTARY MATERIALS TABLES


