# Supplementary material for: Prevalence and time trends of spina bifida in fourteen cities located in the Liaoning province of northeast China, 2006-2015
Source: Oncotarget. 2017 Jan 27;8(12):18943–8. doi: 10.18632/oncotarget.14848 (PMC5386660; doi:10.18632/oncotarget.14848)
Supplement: Supplementary file 1 [file oncotarget-08-18943-s001.pdf]

Prevalence and time trends of spina bifida in fourteen cities located in the Liaoning province of northeast China, 2006–2015

Supplementary Materials

Supplementary Table 1: The number of live births in each city in Liaoning province, 2006–2015

City	Year										Overall
	2006	2007	2008	2009	2010	2011	2012	2013	2014	2015	
Liaoning Province	306734	341432	330414	321353	307826	304079	353108	321171	364400	298437	3248954
Shenyang	52256	61108	59196	59200	57521	58335	69721	67854	80997	65118	631306
Dalian	38744	46652	48309	47900	48774	50490	62324	58722	71178	57641	530734
Anshan	29270	31305	29647	27721	25184	25603	28790	25855	36171	20798	280344
Fushun	11661	12997	12314	12337	11638	11556	12942	12016	12845	10138	120444
Benxi	8620	9435	8759	8842	8696	8261	9440	8700	9857	7627	88237
Dandong	15710	15725	14836	14274	13894	14038	15895	15111	17718	14278	151479
Jinzhou	24293	24261	23149	22342	21255	20098	22559	20860	16137	16985	211939
Yingkou	16987	18924	19667	19070	17947	18484	21309	14224	21684	16515	184811
Fuxin	14158	14142	13353	13322	12370	11800	13050	9662	9121	11752	122730
Liaoyang	12888	15039	13754	13200	12331	11386	13296	11702	12747	9251	125594
Panjing	9887	9669	10134	9009	8800	8867	10362	9644	8276	9197	93845
Tieling	21263	20298	21456	19854	18421	16945	18938	14960	17389	15269	184793
Chaoyang	28669	30980	31168	30574	27837	27207	31236	29919	30646	26083	294319
Huludao	22328	30897	24672	23708	23158	21009	23246	21942	19634	17785	228379

Supplementary Table 2: The prevalence of spina bifida in each city in Liaoning province, 2006–2015 (per 10,000 births)

City	Year										Overall
	2006	2007	2008	2009	2010	2011	2012	2013	2014	2015	
Liaoning Province	9.62	9.52	7.57	6.85	6.72	6.54	5.21	4.42	3.79	2.31	6.25
Shenyang	14.74	16.36	4.90	6.59	6.08	5.31	4.73	4.72	2.96	3.07	6.65
Dalian	5.16	5.14	7.45	3.97	1.03	2.97	3.21	1.87	3.51	1.39	3.45
Anshan	5.81	3.51	0.67	3.61	2.78	3.52	2.08	2.32	0.83	1.44	2.64
Fushun	12.01	7.69	11.37	8.11	5.16	6.92	6.18	0.83	3.11	2.96	6.48
Benxi	5.80	6.36	11.42	5.65	3.45	8.47	1.06	6.90	2.03	1.31	5.21
Dandong	5.09	3.18	4.72	2.80	5.04	4.27	3.77	2.65	2.82	0.00	3.43
Jinzhou	10.70	11.13	6.48	8.50	4.70	7.96	3.55	1.92	5.58	2.36	6.51
Yingkou	1.18	7.40	8.14	3.67	5.01	5.41	6.57	1.41	3.23	0.00	4.38
Fuxin	14.13	16.97	8.99	6.76	15.36	11.02	9.96	2.07	4.39	2.55	9.70
Liaoyang	6.98	8.64	6.54	6.06	6.49	6.15	4.51	1.71	6.28	3.24	5.81
Panjing	4.05	7.24	2.96	5.55	6.82	2.26	4.83	4.15	2.42	1.09	4.16
Tieling	5.17	4.93	9.79	3.02	8.14	5.90	4.75	3.34	3.45	1.31	5.14
Chaoyang	16.74	17.43	15.72	19.62	18.32	15.07	10.24	14.04	9.46	5.75	14.30
Huludao	15.23	6.47	10.94	8.01	11.23	11.42	9.89	9.57	5.09	3.37	9.20
